# Évaluation de la satisfaction des patients dans le service de cardiologie du CHU Yalgado Ouedraogo

**DOI:** 10.11604/pamj.2017.28.267.13288

**Published:** 2017-11-27

**Authors:** Aristide Relwendé Yameogo, Georges Rosario Christian Millogo, Arlette Flore Palm, Joel Bamouni, Germain Dakaboué Mandi, Jonas Koudougou Kologo, André Koudnoaga Samadoulougou, Patrice Zabsonre

**Affiliations:** 1 Service de Cardiologie du Centre Hospitalier Universitaire Yalgado Ouédraogo, Ouagadougou, Burkina Faso; 2 Unité de Formation et de Recherches en Sciences de la Santé (UFR/SDS), Université de Ouagadougou, Burkina Faso; 3 Centre Hospitalier Universitaire Régional de Ouahigouya, Ouahigouya, Burkina Faso

**Keywords:** Satisfaction, évaluation, patients, cardiologie, Burkina Faso, Satisfaction, evaluation, patients, cardiology, Burkina Faso

## Abstract

**Introduction:**

La satisfaction des patients est une composante importante de l'évaluation de la qualité des soins. Les patients sont aujourd'hui des partenaires de soins avec les médecins. Cette nouvelle relation mérite d'être d'évaluée. L'objectif de notre étude était d'évaluer la satisfaction des patients hospitalisés dans le service de Cardiologie du Centre Hospitalier Universitaire Yalgado Ouédraogo.

**Méthodes:**

Il s’agissait d’une étude transversale descriptive à passage unique de l’ensemble des patients hospitalisés du 1er Janvier au 30 Juin 2014. Le questionnaire SAPHORA adapté à notre contexte a été administré pour cette étude. Les scores et les taux de satisfaction ont été calculés en fonction des paramètres étudiés.

**Résultats:**

Durant notre période d’étude, nous avons colligé 235 patients. Le délai moyen d’hospitalisation était d’environ 10 jours. Nous avons noté 125 (53,2%) sujets de sexe masculin soit sex ratio était de 1,1. Trente-deux pour cent (n = 75) des patients étaient non scolarisés. Les fonctionnaires représentaient 24,3% (n = 57) de notre population d'étude. L’âge moyen de notre échantillon était de 50,7 ans. Les patients de plus de 65 ans représentaient 25,6% de la population. Les patients provenaient des urgences médicales dans 113 cas (48,1%). Vingt un patients (8,9%) avaient un antécédent d'hospitalisation dans le service de cardiologie. La cardiomyopathie dilatée était le diagnostic noté en hospitalisation dans 75 (32%) des cas. Le score global de satisfaction des patients pris en charge dans l’unité d’hospitalisation dans le service de cardiologie était de 78,3%. le score de satisfaction de l’accueil était de 68,1% et celui du confort du patient 65,8%. Le score pour la qualité de soins et l'organisation de la sortie étaient respectivement de 84,7% et 84,5%. Les suggestions d'amélioration des patients portaient sur le confort du séjour dans 99 (42,1%), et l'identification du personnel dans 176 (74,9%) des cas . Conclusion: L'évaluation de la satisfaction est peu fréquente dans notre pays. Elle est de plus en plus fréquente dans les pays occidentaux avec des outils communs et validés. Elle constitue un aspect important que nos hôpitaux doivent inscrire pour une démarche qualité en vue d’une accréditation.

## Abstract (en)

**Introduction:**

Patients' satisfaction is an important component of health care quality evaluation. Patients and physicians are now care partners. This new relationship deserves to be evaluated. Our study aimed to evaluate the satisfaction of patients hospitalized in the Department of Cardiology at the University Hospital Yalgado Ouedraogo.

**Methods:**

We conducted a cross-sectional descriptive study with a single data collection phase of all the patients hospitalized from 1 January to 30 June 2014. We administered SAPHORA questionnaire adapted to suit our context. The scores and the satisfaction rates were calculated according to the studied parameters.

**Results:**

During the study period we collected data from 230 patients. The mean hospitalization time was approximately 10 days. 125 (53.2%) men were enrolled in the study, sex ratio was 1.1. 32% (n = 75) of patients were unschooled. Public servants accounted for 24.3% (n = 57) of our study population. The average age of our sample was 50.7 years. Patients over the age of 65 years accounted for 25.6% of the study population. 113 (48.1%) patients had been admitted as medical emergencies. 21 patients (8.9%) had a history of hospitalization in the Depatment of Cardiology. Dilated cardiomyopathy was the diagnosis made during hospitalization in 75 (32%) cases. The overall score of satisfaction of the patients treated in the Department of Cardiology was 78.3%. Satisfaction score on hospital admission was 68.1% and on patients' comfort was 65.8%. Satisfaction score on health care quality and on hospital discharge planning was 84.7% and 84.5% respectively. Patients' suggestions for improvement were based on comfort during hospital stay in 99 (42.1%) cases and on staff identification in 176 (74.9%) cases.

**Conclusion:**

The evaluation of the satisfaction is infrequent in our country. It is becoming increasingly frequent in western countries using common and validated tools. It is an important aspect that our hospitals should include in order to increase quality approach to accreditation.

## Introduction

Selon l'Organisation Mondiale de la Santé: « L'évaluation de la qualité des soins est une démarche qui permet de garantir à chaque patient des actes diagnostiques et thérapeutiques assurant le meilleur résultat en terme de santé, conformément à l'état actuel de la science médicale, au meilleur coût pour le meilleur résultat, au moindre risque iatrogène et pour sa plus grande satisfaction en termes de procédures, de résultats et de contacts humains à l'intérieur du système de soins » [1]. La satisfaction du patient est une composante de l'évaluation de la qualité des soins. La mesure de la satisfaction des patients s'inscrit dans un contexte global de montée de la place de l'usager dans l'organisation du système de santé et la place croissante des démarches qualités. Le service rendu au client et donc son appréciation tiennent une place centrale [2]. Les patients sont aujourd'hui des partenaires actifs des soins qui leur sont proposés et leur satisfaction est considérée comme un indicateur de la qualité des soins [3]. Le recueil de leur point de vue est nécessaire pour évaluer la qualité d'un système de soins au même titre que les considérations techniques que pourront livrer les professionnels de santé [2]. La qualité de cette nouvelle relation entre le patient et son médecin et de la satisfaction qu'en exprime le patient doit être évaluée afin d'apporter une amélioration continue [3]. L'intérêt porté à la qualité des soins n'a cessé de croître ces dernières années. La majorité des établissements de santé la place au c'ur de leurs priorités notamment avec la création des réseaux de soins où les patients sont des acteurs de leur prise en charge [4]. Les raisons en sont multiples: l'évolution constante et rapide de la médecine grâce aux progrès scientifiques, des revendications de plus en plus nombreuses concernant l'accessibilité ainsi que la qualité des soins et les nouvelles technologies, tout en exigeant des garanties de sécurité [4].

Toutes ces raisons conduisent à prendre en compte les sentiment de satisfaction exprimé par le patient. Elles prennent d'autant plus de poids, dans le contexte du plus grand degré d'information des personnes sur les maladies et les possibilités de traitement ou de prévention existantes [2]. La mesure de la satisfaction s'appuie sur des moyens multiples: les plaintes, les questionnaires de sortie mais aussi des enquêtes spécifiques ou générales menées à l'initiative des établissements de santé [2]. Bien qu'elle soit présente dans les textes réglementaires un peu partout dans le monde, la satisfaction du patient est un élément qui reste encore trop négligé par les praticiens [5]. La mesure de la satisfaction des patients permet de décrire la prise en charge du point de vue patient, d'identifier les problèmes et d'y apporter dans le cas échéant des solutions [3]. L'analyse de la littérature a montré que les études réalisées sur la satisfaction des patients aux Etats-Unis, dans les pays nordiques et en Europe avaient un taux compris entre 68 et 98% en 2004 [6]. En Afrique, les études sont moins nombreuses. La Tunisie notait un taux de satisfaction de 51% en 2005 [7]. En Afrique de l'Ouest, elles sont encore plus rares. Dans notre pays le Burkina Faso quelques études ont noté des taux de satisfaction compris entre 52.7 et 94.12% [8-13]. Néanmoins, il n'existe pas un système de suivi de la satisfaction des patients, ni un texte réglementaire imposant sa mesure au niveau des structures sanitaires. Notre étude était la première expérience dans l'évaluation de la satisfaction des patients en Cardiologie du Centre Hospitalier Universitaire-Yalgado Ouédraogo (C.H.U-Y.O). Elle avait pour objectif d'évaluer la satisfaction des patients pris en charge dans l'unité d'hospitalisation dans le service de Cardiologie du C.H.U-Y.O afin d'améliorer la qualité des soins et des services.

## Méthodes

**Type et période d´étude:** Il s'agissait d'une étude transversale descriptive à passage unique qui s'est déroulée du 1^er^ Janvier au 30 Juin 2014.

**Service de cardiologie:** Le service de cardiologie est un service du département de Médecine du Centre Hospitalier Universitaire-Yalgado Ouédraogo. Il comprend plusieurs unités: l'unité de consultation externe, de suivi et d'exploration fonctionnelle des patients; l'unité du bloc opératoire et d'électrophysiologie fonctionnelle depuis 2010 dont les activités sont surtout centrées sur la pose de pacemaker; l'unité d'hospitalisation et l'unité de soins intensifs non fonctionnelle depuis les inondations de 2009. L'unité d'hospitalisation de la cardiologie du C.H.U.-Y.O. comporte six salles d'hospitalisation avec une capacité d'accueil de 30 lits dont 2 salles de 2 lits chacune pour la 1ère catégorie avec un tarif de 4500 francs CFA /jour; 2 salles de 3 lits chacune pour la 3èmecatégorie à 1000 francs CFA/jour et 2 salles de 8 lits chacune pour la 4ème catégorie à 500 francs CFA /jour. L'unité de soins ne dispose pas de 2ème catégorie. Seules les deux salles de la 1ère catégorie disposent chacune de commodités sanitaires. Chaque médecin cardiologue a un jour de consultation des malades venus pour contrôle ou en consultation externe et un jour de visite médicale aux malades hospitalisés. Tous les vendredis, la visite médicale aux malades est conjointe et elle est faite par l'ensemble des médecins. Un programme d'astreinte des médecins cardiologues prenant en compte les week-ends et les jours fériés est dressé en début de chaque mois. Les médecins en spécialisation de cardiologie assurent les gardes et les permanences dans le service. La visite des malades est donc assurée tous les jours de la semaine. Un médecin en spécialisation de cardiologie assurait tous les matins la visite et la contre visite des patients présentant des pathologies cardiaques dans le service des urgences médicales. Le transfert du patient se voyait ainsi donc relativement plus aisé. Les patients provenant directement des autres services pouvaient être hospitalisés à tout moment de la journée par le médecin en spécialisation de permanence ou de garde.

**Population et taille d'étude:** La population d'étude correspondait à l'ensemble des patients hospitalisés dans le service de cardiologie durant notre période d'étude. Les critères d'inclusion étaient: tous les patients d'âge supérieur ou égal à 18 ans; patients hospitalisés pour une pathologie cardio-vasculaire, dont la durée du séjour était d'au moins 48h; patients dont la destination de sortie était le domicile; patients ayant consenti à participer à l'étude. les critères de non-inclusion étaient: patients sortis contre avis médical ou transférés dans un autre service; les patients décédés. La taille de l'échantillon a été calculée à partir de la formule de l'échantillonnage d'un sondage est le suivant:

$$n=\frac{Np\left(1-p\right)}{\frac{d^{2}}{Z_{1-a/2}^{2}2}\left(N-1\right)+p\left(1-p\right)}$$

N = nombre de patients hospitalisés par an dans le service à l'année n-1 (2012) = 600. d = largeur de l'intervalle 5% α = risque d'erreur 5%. p = taux de satisfaction 50%. Ce taux a été choisi car nous avions pas de donner sur la satisfaction des patients dans la prise en charge des maladies cardiovasculaires. Cette formule nous donnait un effectif de 235 patients à collecter. L'échantillon des patients a été constitué au fur et à mesure des sorties d'hospitalisation. Il n'y a pas eu de tri préalable par rapport au sexe, la provenance géographique et au niveau socio-économique. Chaque patient était libre d'accepter ou de refuser l'interview. La durée moyenne de l'interview était de 10 minutes.

**Questionnaire de satisfaction:** Nous avons utilisé un questionnaire SAPHORA-MCO version 2009 adapté pour l'évaluation de la satisfaction des patients en médecine, en chirurgie et en obstétrique. Nous avons réalisé un pré test avec le questionnaire complet qui nous a permis de relever certaines difficultés: l'unité d'hospitalisation de la cardiologie ne disposait pas d'un service administratif; nous ne réalisons pas d'intervention chirurgicale dans le service; un tiers de nos patients étaient non scolarisés; tous les patients interrogés ne savaient pas distinguer les différentes personnes travaillant dans le service; les chambres d'hospitalisation ne disposent pas de téléviseur ni de téléphone et seules les chambres de 1ère et de 3ème catégorie disposent de climatisation; les patients ne bénéficient pas d'aide du personnel de soins pour les activités de la vie courante et la prestation repas est indépendante du service de cardiologie. Après le pré test, nous avons adapté le questionnaire à notre contexte en supprimant les questions qui ne pouvaient pas être renseignées ou non adaptées à notre contexte. Ce questionnaire a été administré en français et traduit simplement en langue pour ceux qui ne comprennaient pas le français avec des niveaux représentés de 1 à 5 (1: très satisfaisant; 2: satisfaisant; 3: mauvais; 4: très mauvais et 5: non concerné).

**Outils de collecte:** Les outils de collecte étaient: les dossiers des patients: ils étaient utilisés pour la collecte des informations sur le malade. Ses antécédents médicaux par rapport aux pathologies cardiovasculaires, le diagnostic de son hospitalisation et sa pathologie antérieure s'il a déjà été hospitalisé dans le service; le registre d'entrée et de sortie; une fiche d'entrevue des patients. Les fiches étaient anonymes afin de rassurer les patients. Les patients étaient informés que les réponses de leur questionnaire n'allaient pas influencer leur prise en charge ultérieure; des appels téléphoniques pour certains patients qui ne souhaitaient pas répondre immédiatement mais consentent à participer à l'étude; l'observation directe des agents de santé pour détecter les dysfonctionnements dans le service; le questionnaire SAPHORA-MCO a été adapté à notre contexte.

**Mode de calcul des indicateurs:** Le taux de satisfaction est calculé pour chaque question: il s'agit du pourcentage de patients ayant choisi la modalité de réponse positive parmi les 5 modalités proposées. Mode de calcul: nombre de patients ayant choisi la modalité de réponse positive sur ensemble des patients ayant donné une réponse de satisfaction à la question. Le score de satisfaction est calculé à partir des réponses à plusieurs questions, ils sont au nombre de 3: "Qualité des soins" (11 questions): accueil dans le service de soins, le temps d'attente, identification des différentes personnes travaillant dans le service, respect de l'intimité, le respect de la confidentialité, information sur le traitement et l'état de santé, amabilité et disponibilité du personnel, délai d'attente aux examens, prise en charge de la douleur, suivi du traitement, satisfaction globale par rapport aux soins; "Confort" (5 questions): confort et propreté de la chambre, bruit dans le service, l'horaire des repas, qualité et quantité des repas, respect des régimes; "Organisation de la sortie" (4 questions): Information sur les médicaments, information sur les activités possibles après la sortie, informations données sur la continuité des soins, les formalités administratives de sortie. Mode de calcul: chaque score est la somme des réponses aux questions positives le composant divisée par le nombre de questions répondues et ramené à 100. Les résultats sont exprimés par des scores de 0 (satisfaction minimale) à 100 (satisfaction maximale).

**Analyses des données:** Les données étaient saisies sur ordinateur et analysées à l'aide du logiciel EPIINFO 7.1.3.0 et le logiciel R.

**Considérations éthiques:** La commission médicale d'établissement de l'hôpital a donné son accord pour la réalisation de l'étude. Les patients ont donnés leur consentement pour la réalisation de l'étude. Le refus de participation à l'étude n'entrainait aucune modification ni sanction à la prise en charge du patient. La confidentialité et l'anonymat des données ont été assurés.

## Résultats

Durant notre période d'étude, nous avons colligé 235 patients dans l'unité d'hospitalisation du service de la Cardiologie du Centre Hospitalier Universitaire Yalgado OUEDRAOGO (C.H.U.-Y.O.). Le délai moyen d'hospitalisation était d'environ 10 ± 14 jours avec des extrêmes de 2 à 45 jours selon la pathologie. Nous avons noté 125 (53.2%) sujets de sexe masculin soit un sex ratio était de 1.1. Trente-deux pour cent (n = 75) des patients étaient non scolarisés. Les fonctionnaires représentaient 24.3% (n = 57) de notre population d'étude. Le Tableau 1 montre les caractéristiques de la population. L'âge moyen de notre échantillon était de 50.7 ± 18.1 ans avec des extrêmes de 18 et 99 ans. Les patients de plus de 65 ans représentaient 25.6% de la population. L'âge moyen des patients de sexe féminin était de 49 ± 1 9.3 avec des extrêmes de 18 et 99 ans. Le Tableau 1 montre la répartition des patients en fonction de la tranche d'age. Les patients provenaient des urgences médicales dans 113 cas (48.1%). La chambre de catégorie 4 était occupée dans 138 (58.7) cas. Le Tableau 2 montre la répartition des patients selon le mode d'entrée en hospitalisation et des chambres d'hospitalisation. Vingt un patients (8.9%) avaient un antécédent d'hospitalisation dans le service de cardiologie. La cardiomyopathie dilatée était le diagnostic noté en hospitalisation dans 75 (32%) des cas. Le Tableau 3 donne la répartition des patients en fonction du diagnostic. Le score global de satisfaction des patients pris en charge dans l'unité d'hospitalisation dans le service de cardiologie était de 78.3%. Le score de satisfaction de l'accueil était de 68.1% et celui du confort du patient 65.8%. Le score pour la qualité de soins et l'organisation de la sortie étaient respectivement de 84.7% et 84.5%. Tous les patients acceptaient recommander le service à leurs parents ou connaissances. Les Tableau 4 et Tableau 4 (Suite) montrent la répartition des patients en fonction des taux de satisfaction par question. Les suggestions d'amélioration des patients portaient sur le confort du séjour dans 99 (42.1%), le silence dans 42 (18.3%) et l'identification du personnel dans 176 (74.9%) des cas.

**Tableau 1:** Caractéristiques de la population (N = 235)

	Fréquence	Pourcentage
Niveau de scolarisation		
Non scolarisé	75	31.9
Primaire	50	21.3
Secondaire	76	32.3
Supérieur	34	14.5
Profession		
Cultivateur/ femme au foyer	93	39.6
Fonctionnaire	57	24.3
Élève / Étudiant	10	04.3
Artisan	28	11.9
Commerçant(e)	43	18.3
Autres	04	01.7
Tranche d'âge en année		
<= 25	14	06.0
[25–35]	48	20.4
[35–45]	51	21.7
[45–55]	29	12.3
[55–65]	36	15.3
> 65	57	24.3

**Tableau 2:** répartition des patients en fonction du mode d'entrée et la chambre d'hospitalisation (N = 235)

	Fréquence	Pourcentage
**Mode d'entrée dans le service**		
Médecin du service	60	25.5
Transfert des urgences	113	48.1
Consultation du service	19	08.1
Directe	43	18.3
**Chambre d'hospitalisation**		
Catégorie 1	28	11.9
Catégorie 3	69	29.4
Catégorie 4	138	58.7

**Tableau 3:** Répartition des patients en fonction du diagnostic d'hospitalisation (N=235)

	Fréquence	Pourcentage
Cardiomyopathie dilatée	75	31.9
Maladie thrombo-embolique veineuse	70	29.8
Valvulopathie	26	11.1
Accident vasculaire cérébral ischémique	10	04.3
Accident vasculaire cérébral hémorragique	09	03.8
Péricardite	13	05.5
Syndrome coronarien aiguë	09	03.8
Cœur pulmonaire chronique	05	02.1
Endocardite aiguë	04	01.7
Cardiothyréose	01	00.4
Cardiopathie rythmique	13	05.5
**Total**	**235**	**100**

**Tableau 4:** Taux de satisfaction des patients en fonction des questions (N=235)

	Très satisfaisant (%)	Satisfaisant (%)	Mauvais (%)	Très mauvais (%)	Non concerné (%)
**Qualité des soins**					
Accueil dans le service de soins	48 (20.4)	182 (77.4)	03 (1.3)	02 (0.9)	
Temps d’attente	49 (20.9)	180 (76.6)	04 (1.7)	02 (0.9)	
Identification des différentes personnes travaillant dans le service	01 (0.4)	20 (8.5)	210 (89.4)	04 (1.7)	
Respect de l’intimité	09 (3.8)	219 (93.2)	07 (3.0)		
Respect de la confidentialité	09 (03.8)	221 (94.0)	05 (2.1)		
Information sur le traitement et l’état de santé	26 (11.1)	83 (35.3)	119 (50.6)	07 (3.0)	
Amabilité et disponibilité du personnel	27 (11.5)	190 (80.9)	16 (6.8)	02 (0.9)	
Délai d’attente aux examens	05 (2.1)	225 (95.7)	04 (1.7)	01 (0.4)	
Prise en charge de la douleur	20 (21.7)	71 (77.2)	01 (1.1)		143 (60.8)
Suivi du traitement	30 (12.8)	201 (85.5)	04 (1.7)		
Satisfaction globale par rapport aux soins	22 (9.4)	211 (89.8)	02 (0.9)		
**Confort du séjour**					
Confort et propreté de la chambre	06 (2.6)	204 (86.8)	23 (9.8)	02 (00.9)	
Bruit dans le service	05 (2.1)	162 (68.9)	62 (26.4)	06 (02.6)	
L’horaire des repas	02 (1.6)	113 (92.6)	07 (5.8)		113(48.1)
Qualité et quantité des repas	02 (1.6)	96 (78.7)	24 (19.7)		113(48.1)
Respect des régimes	01 (1.2)	74 (87.0)	10 (11.8)		150 (63.8)
**Organisation de la sortie**					
Information sur les médicaments	29 (12.3)	206 (87.7)			
Information sur les activités possibles après la sortie	26 (11.1)	70 (29.8)	139 (59.1)		
Informations données sur la continuité des soins	31 (13.1)	202 (86)	02 (0.9)		
Les formalités administratives de sortie	03 (1.3)	227 (96.6)	05 (2.1)		

## Discussion

Le score de satisfaction de l'accueil est de 68.1%. Ce score a été rabaissé à cause du mauvais taux de satisfaction de l'identification du personnel dans le service (9%). L'accueil du patient et de sa famille demeure un facteur primordial. L'accueil est plus qu'un acte banal de la vie quotidienne. L'arrivée d'un malade dans un service de soins est particulier. C'est pour lui un temps fort, un moment d'imprégnation où il est sensible et vulnérable et où il a besoin de se raccrocher à quelqu'un. L'accueil se confirme ensuite au cours de son séjour, à travers le soutien qui lui est apporté dans ses difficultés, par l'écoute et la compréhension que le personnel lui manifeste, autre élément majeur de la qualité des soins. Tout acte de soin est un acte relationnel et l'aspect de la communication lui donne la forme d'un acte humain par excellence. C'est ce qui fait la différence entre « donner des soins » et «prendre soins». «Donner des soins» représente des gestes banals, aux aspects techniques, bien sûr fort importants, mais « prendre soin » de quelqu'un possède un sens beaucoup plus profond. Cela suppose une implication personnelle des qualités de soins et d'intelligence bien différentes. Il inclut des considérations éthiques, des manifestations d'empathie, des stratégies organisationnelles adaptées aux conditions et aux besoins du malade et de sa famille. La nécessité s'en fait surtout sentir dans des situations critiques. C'est pourquoi ce soutien est une composante essentielle de la qualité des soins [14]. En effet, dans le service de cardiologie, le score pour la qualité de soins et l'organisation de la sortie étaient respectivement de 84.7% et 84.5%. Ces résultats sont le fruit de l'organisation du service de cardiologie. Les médecins en spécialisation voient les patients aux urgences et informent le service de l'arrivée des nouveaux patients. Toute l´équipe prépare les salles et le matériel nécessaire en fonction de la gravité de la maladie. A la sortie des patients, il est remis un carnet de santé au patient avec son ordonnance de sortie et le prochain rendez vous pour le contrôle avec son cardiologue, assurant ainsi la continuité des soins.

La propreté et le confort de la chambre étaient satisfaisants dans 89.4%. En effet, toute l'unité d'hospitalisation est nettoyée tous les matins par une équipe de nettoyage de l'hôpital. Néanmoins, ce nettoyage reste insuffisant car il est fait à la hâte, très tôt le matin ce qui dérange les patients. De plus, après ce nettoyage matinal, il n'y a plus de nettoyage toute la journée. En cas de besoin, il est réalisé par les accompagnants ou les filles de salle qui sont en nombre insuffisant dans le service. Les patients ne disposaient pas de poubelle individuelle mais d'une poubelle commune à proximité du service. Le confort et la propreté dépendent également du niveau socio-économique de la population étudiée, de ce fait ils restent très subjectifs. Le niveau de bruit était jugé satisfaisant par 71% des patients. Les visiteurs venaient en nombre important et oubliaient très souvent de mettre les téléphones sous vibreur. La promiscuité des patients participait à la difficulté d'obtenir un calme dans les salles. Malgré la sensibilisation de limiter les visites, les accompagnants restent intraitables à ce sujet. Cela nécessite de mettre en place d'autres mécanismes pour la gestion des accompagnants. Le score de satisfaction globale de la prise en charge était de 78.3%. Des études dans les services au Burkina Faso ont trouvé des scores entre 52 et 94% [8-12]. Mais ces scores doivent être interprétés avec caution car ils n'ont pas été calculés avec des outils validés mais des outils adaptés par les enquêteurs. Le niveau élevé de satisfaction montrait que la majorité des patients jugeaient positivement le mode d'organisation de l'unité d'hospitalisation de la cardiologie. Néanmoins, ce niveau élevé doit être interprété avec précaution. Le mécontentement n'est exprimé que quand un événement grave se produit et en ce sens une réponse positive dans une étude de satisfaction ne veut pas toujours dire que la prise en charge était bonne, mais simplement que rien de grave ne s'est produit [15].

En ce sens, il paraît important de porter une attention particulière aux questions pour lesquelles les patients ne sont pas complètement satisfaits. Mais un taux de satisfaction aussi élevé est-il vrai. Cette interrogation est légitime et témoigne d'un désir de savoir vraiment ce que pensent les patients, au-delà de l'envie d'être perçu comme un « bon médecin » et la volonté de se confronter à la critique des patients. Ce taux général atteint pose plusieurs questions, notamment des biais possibles [16]. La réalisation des interviews à la sortie d'une hospitalisation et le fait de mener ces interviews en direct pouvaient comporter des biais. Mais probablement moins qu'une enquête écrite qui poserait des problèmes de langage et de l'utilisation de l'écrit, 31.9% de nos patients étant non scolarisée. Réaliser des interviews à distance de l'hospitalisation, c'est-à-dire à domicile, se heurtait à des difficultés de réalisation (certains vivant en zone semi rurale et rurale, certains patients n'ayant pas d'adresses de contact), dans l'interprétation des résultats et le biais de mémoire (certains patients ne se rappelleront pas tous les détails du séjour). Le fait que le questionnaire était administré par un agent de santé pourrait être un biais. Les patients ayant une crainte de critiquer ouvertement le personnel de santé et de ce fait de parler de leurs insatisfactions. Le contexte socioculturel des patients pourrait également constituer un biais. En effet, la santé retrouvée constituait déjà pour certains patients une source de satisfaction et influençait les réponses aux questions. Cela était perceptible chez les patients provenant des zones rurales. Ceux-ci étant à la recherche de l'amélioration de leur santé et n'ayant pas d'exigences concernant la qualité des soins. Les autres biais possibles pouvaient être le fait que le personnel de santé était au courant de l'étude. Donc, qu'il ait eu de manière consciente ou non une adaptation de l'attitude du personnel de santé face aux patients. Une des limites de notre étude était le caractère tranché et fermé des questions qui ne permettaient pas au patient de s'exprimer et de faire des commentaires librement.

## Conclusion

La mesure de la satisfaction des patients est aujourd'hui une préoccupation générale de tous les établissements de santé. Elle constitue une source d'informations pour l'amélioration de la qualité de soins. Les résultats obtenus dans notre étude sur la satisfaction des patients au sortir de leur hospitalisation en Cardiologie sont encourageants. Cependant, plusieurs aspects d'insatisfaction ont été relevés concernant l'identification du personnel de santé, la communication, le niveau sonore et la restauration. Ces aspects peuvent nettement être améliorés si des mesures correctives sont entreprises. La diversité des personnes hospitalisées et de leur parcours génèrent des attentes difficilement exprimées. La notion de satisfaction reste donc subjective et relative. Peu fréquente dans notre contexte, elle est de plus en plus en évaluation dans les pays occidentaux avec des outils communs et validés. Les études de satisfaction constituent donc un aspect important que nos hôpitaux doivent inscrire pour une démarche qualité.

### Etat des connaissances actuelle sur le sujet

- L'évaluation de la satisfaction des patients est un indicateur important pour la qualité des soins dans les hôpitaux;
- La satisfaction des patients n'est pas un indicateur pris en compte dans l'évaluation de la qualité des soins dans nos hôpitaux.

### Contribution de notre étude à la connaissance

- L'identification des agents de santé est un élément important pour les patients;
- Le confort de séjour des patients doit être amélioré dans nos hôpitaux;
- Une importance doit être accordée aux points d'insatisfaction car bien que faible constituent des éléments importants d'évaluation.

## Conflits d’intérêts

Les auteurs ne déclarent aucun conflit d'intérêts.
